# Effects of rosiglitazone/PHBV drug delivery system on postoperative fibrosis in rabbit glaucoma filtration surgery model

**DOI:** 10.1080/10717544.2019.1648590

**Published:** 2019-08-07

**Authors:** Feng Zhang, Ke Liu, Zheng Pan, Mengdan Cao, Dengming Zhou, Hairong Liu, Yuting Huang, Xuanchu Duan

**Affiliations:** a Department of Ophthalmology, The Second Xiangya Hospital, Central South University, Changsha, Hunan Province, China;; b College of Materials Science and Engineering, Hunan University, Changsha, Hunan, China;; c Aier School of Ophthalmology, Central South University, Changsha, Hunan, China;; d Changsha Aier Eye Hospital, Changsha, Hunan, China

**Keywords:** Drug delivery system, PHBV, rosiglitazone, glaucoma filtration surgery

## Abstract

The aim of this study is to investigate the effects and toxicities of poly(3-hydroxybutyric acid-*co*-3-hydroxyvaleric acid) (PHBV)-loading rosiglitazone on preventing scar formation after glaucoma filtration surgery (GFS) in the rabbit model. Rosiglitazone/PHBV drug delivery system was prepared via electrospinning. Release behavior of RSG/PHBV membrane was evaluated by high-performance liquid chromatography. The different concentration membranes were implanted under the conjunctiva of the rabbit’s eyes (RSG/PHBV groups). Also, MMC-soaked sponges were placed under the conjunctiva of the eyes (positive group) for 3 min. Intraocular pressures and bleb features were then assessed for 4 weeks postoperative. Bleb sections were stained with HE, Masson’s trichrome and α smooth muscle action (αSMA) immunohistochemistry. The protein expression of collagen I, αSMA, and connective tissue growth factor in the bleb area were then quantified. The following results were observed: (1) the concentration of rosiglitazone would not affect the morphology of RSG/PHBV membrane. (2) RSG/PHBV membrane would effective and safety prevent the formation of fibrosis after GFS in the rabbit model. Implantation of RSG/PHBV membrane prevents scar formation after GFS. What’s more, it ameliorated toxicity to conjunctiva and cornea compared with the placement of MMC. The RSG/PHBV membrane would be a more effectivity and safer strategy than MMC.

## Introduction

Glaucoma, the most common cause of irreversible blindness in the world, has a progressive optic neuropathy and a corresponding visual field loss. Topical eye drops of lowering intraocular pressure (IOP) is the first-line treatment for glaucoma (Ozdemir et al., 2017). However, glaucoma surgical treatment would applied in the patients whose IOP cannot controlled under the treatments of medicine or laser therapy. A successful glaucoma filtration surgery (GFS) depends on the extent and quality of wound modulation after operation (Seibold et al., 2012). Mitomycin C (MMC) and 5-fluorouracil (5-FU) are the most effective and popular antifibrosis drugs for GFS (Lockwood et al., 2013). Although these have proven efficacy in wound modulation, they also have some serious complications such as corneal toxicity, wound leak, hypotony, scleral ischemia and even infectious endophthalmitis (Anand & Khan, 2009; Vahedian et al., 2017). Therefore, an effective and safer approach to inhibiting fibroblast proliferation after GFS is currently greatly needed.

Rosiglitazone, specific peroxisome proliferator-activated receptor-γ(PPAR-γ) ligands, could attenuate activation of human Tenon’s fibroblasts induced by transforming growth factor-β1 according to our previous research (Luo et al., 2014). In this study, the rosiglitazone drug delivery system (DDS) would be made to prevent the formation of scarring after GFS. Among polymeric NPs, poly(3-hydroxybutyric acid-*co*-3-hydroxyvaleric acid) (PHBV) constitutes a promissory alternative, more accessible and cheaper than similar polymers such as poly(lactic-*co*-glycolic acid) (Peñaloza et al., 2017). PHBV, a biodegradable and biocompatible polyester, is produced by bacteria or eukaryotic cells and the metabolites of PHBV are CO_2_ and H_2_O. Therefore, it can be synthesized in large scales (Kuntanoo et al., 2015).

Electrospinning is broadly used for electrostatic fiber formation which utilizes electrical forces to produce polymer fibers with diameters ranging from 2 nm to several micrometers (Bhardwaj & Kundu, 2010). Nanofiber mats have high functional characteristics and increasing surface area which is associated with drug dissolution and release. So, the technology of electrospinning has been used by a number of researchers for DDS (Hu et al., 2013; Nagy et al., 2013; Hamori et al., 2014; Fathollahipour et al., 2015; Mei et al., 2016; Li et al., 2017). Also, electrospinning would be used in the combination of rosiglitazone with PHBV in our study.

## Materials and methods

### Materials

The assays include chloroform (TCM), dimethylformamide (DMF), dimethyl sulfoxide (DMSO) and PHBV, which were purchased from Sigma (USA). Rosiglitazone was obtained from BioVision (USA). Ammonium formate came from Fisher scientific (USA). Acetonitrile was obtained from Merck Millipore (Germany). Filed emission scanning electron microscope SU8010 was obtained from Hitachi (Tokyo, Japan). LC-20A high-performance liquid chromatography (HPLC) were obtained from Shimadzu equipped with an autosampler (Model SIL-20A), a column temperature controller compartment (CTO-10AS) and ultraviolet detector (SPD-M20A). Diamonsil C18 column (5 μ 250 × 4.6 mm) were purchased from Dikma Technologies Inc., China. PH meter came from Denver Instrument, USA. TonoVet were obtain from Icare, Finland. In the vivo experiments, the assays include pentobarbital sodium (30 mg/kg) (Solarbio, Beijing, China), Benoxil (Santen, Japan), Tobramycin Dexamethasone Eye Ointment (Alcon, USA), 0.5% Levofloxacin Eye Drops (Santen, Japan), 4% Paraformaldehyde (Solarbio, Beijing, China), suture line (10-0 nylon; Alcon; USA), 8-0 Vicryl suture (Ethicon; USA), Normal Saline (Second Xiangya Hospital, Changsha, China), Mitomycin C (Sigma, USA), anti-Collagen I antibody (Abcam, UK), anti-α-SMA antibody (Abcam, UK), and anti-connective tissue growth factor (CTGF) antibody (Abcam, UK).

### Preparation of rosiglitazone/PHBV membrane

**Figure 1.**
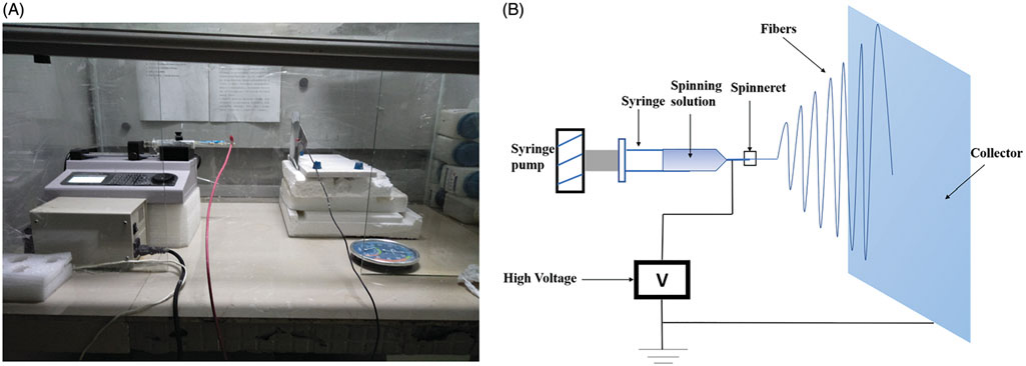
(A) The photograph of the electrospinning apparatus. (B) The drawing of the electrospinning apparatus.

The electrospinning process is governed by many parameters including solution parameters, process parameters, and ambient parameters. We can get fibers of desired morphology and diameters by properly manipulate these parameters. PHBV (500 mg) was dissolved in 9 mL of Chloroform (TCM). The solution was continuous stirring by a homogenizer in the room temperature and then dissolved by the Ultrasonics processor in 60 °C for 8 min. Afterwards, 1 mL of DMF was added to the PHBV-TCM solution and stirring was continued for an additional 5 min. The solution of 5% (w/v) PHBV-TCM/DMF (9/1) was readied. At the same time, rosiglitazone (RSG) was dissolved in DMSO (100 μL). There are four different concentration of RSG/DMSO solution: 0, 0.5, 5, 50 mg/mL. Then, mixed the solution of PHBV-TCM/DMF and RSG/DMSO. Therefore, the final concentration of RSG in the spinning solution were 0, 0.05, 0.5, 5 mg/mL. The prepared spinning solution was placed in a 20 mL injection syringe and extruded at the speed of 5 mL/h by a syringe pump (LSP01-1A, Longer Pump, UK), and a high voltage (15 kV) was applied between the spinning needle (26G) and ground collector which was covered with aluminum foil with the distance of 15 cm (Figure 1). The room temperature was 20–24 °C and relative humidity (RH) was 20%–40% during the experiment. Finally, the RSG/PHBV membranes were removed from collector and dried in the room temperature for 3 days.

### Field emission scanning electron microscopy

Surface morphological structures of RSG/PHBV membranes were observed by a field emission scanning electron microscope (SU8010, Hitachi, Tokyo, Japan). The applied accelerating voltage and working distance were 3.0 kV and 11–12 mm, respectively.

### *In vitro* drug release studies

In our present experiment, four different concentration of RSG in RSG/PHBV membranes were divided into four groups accordingly: Group A (0 mg/mL), Group B (0.05 mg/mL RSG), Group C (0.5 mg/mL), Group D (5 mg/mL). The RSG/PHBV membranes were cut into pieces (3 cm × 3 cm). Then the membranes were placed immediately into 1 ml of physiological saline and supernatant solution was collected to detect the concentration of rosiglitazone at different times by HPLC. The mobile phase was composed of a mixture of 20 mM formic acid aqueous solution and acetonitrile (45:55). The flow rate was set at 1.0 mL/min and the injection volume was 20 μL. The temperature of column was 40 °C and the chromatograms were detected at 247 nm.

### Animals models and groups

Adult male rabbits (New Zealand white rabbits, 2.5–3.5 kg) were used in the study. The rabbits were adapted for 1 week before experimentation. Approval for this study was obtained from the Second Xiangya Hospital of the Center South University. All animals were treated in accordance with the ARVO Statement for the Use of Animals in Ophthalmic and Vision Research. The IOP of both eyes was recorded with a Tonovet (icare). The animals were anesthetized with an intravenous injection of pentobarbital sodium (30 mg/kg) (Solarbio, Beijing, China). At first, an eyelid speculum was inserted to expose surgical field and the eye was fixed with a corneal traction suture (7-0 Vicryl; Ethicon; USA). Then, a half-thickness, limbal-based, 4 × 4 mm scleral flap was made anterior to the limbus. The scleral flap was sutured with two suture line (10-0 nylon; Alcon; USA) and the RSG/PHBV membrane (3 cm × 3 cm) was fixed in the top of scleral flap with 10-0 nylon suture line. The conjunctiva was closed with 8-0 Vicryl suture (Ethicon; USA) in a continuous fashion. In order to reduce inflammation of surgery eye, 0.5 mL dexamethasone was injected in the subconjunctiva. At last, appropriate amount of Tobramycin Dexamethasone Eye Ointment (Alcon, USA) (one time daily) and Tobradex eye drops (four times daily) were used on the surface of surgery eyes in the first week. All surgeries were performed by the same researcher.

The rabbits were randomized to five treatment groups: (1) Group A (vehicle control) (*n* = 6): this group was using 0 mg/mL RSG/PHBV membrane during the operation; (2) Group B: this group was using 0.5 mg/mL RSG/PHBV membrane intraoperatively; (3) Group C: this group was using 5 mg/mL RSG/PHBV membrane intraoperatively; (4) Group D: this group was using 50 mg/ml RSG/PHBV membrane during the surgery. (5) mitomycin C-treated group (*n* = 6): this positive control group was using 0.4 mg/mL MMC cotton pat intraoperatively for about 3 min; We would track and follow up from postoperative day 1 to day 28. The bleb appearance, survival, IOP and complications were examined. In addition, these rabbits were killed on postoperative day (POD) 14 or 28 by an intravenous injection of overdose pentobarbital sodium and compared the scaring by performed immunohistochemistry and Western blot analysis. There are no intraoperative complications occurred in any rabbits, so no rabbits were excluded from this study.

### IOP measurement and bleb scores

The IOP of rabbits were measured with a Tonovet (Vantaa, Finland) at 10:00 O’clock every day. The final IOP measurements we record is the average of three IOP measurements. All measurements were performed by the same researcher. The bleb’s scoring was conducted according to Indiana bleb grading appearance scale (IBAGS) set by Picht & Grehn (1998). Bleb height, extent, vascularity, and leakage with the Siedel test were evaluated under a slit lamp. The score of a bleb was the mean value of scores given by three experienced graders. Furthermore, a slit-lamp experiment was performed to examine the complications (hyperemia, chemosis of the conjunctiva, corneal toxicity, wound leak, blebtitis, bleb leakage, endophthalmitis) in these four groups.

### Histology and immunohistochemistry

The tissues of operation sites (bleb, conjunctiva, Tenon’s capsule and sclera) were fixed in 4% paraformaldehyde and embedded in paraffin. Thereafter, hematoxylin and eosin (H&E) staining, Masson’s trichrome staining and immunostaining of αSMA were performed. Results were observed and imaged by light microscopy (Nikon Eclipse CI, Japan).

### Western blot analysis

Samples of operation sites were washed thrice using ice-cold PBS followed by extracting in cold radioimmunoprecipitation assay (RIPA) lysis buffer (Beyotime, Shanghai, China), which contained 1% protease inhibitor cocktail (Sigma-Aldrich, St. Louis, MO) and 1% phosphatase inhibitor (Sigma-Aldrich). Protein concentrations were determined using a BCA protein assay kit (Beyotime, Shanghai, China). An equal amount of protein was subjected to gel electrophoresis and transferred to poly(vinylidene fluoride) membranes (GE, Pittsburgh, PA) followed by blocking in 5% nonfat dried milk in TBST at room temperature for 1 h and incubated with primary antibodies overnight at 4 °C. After washing with TBST five times for 30 min, the membranes were incubated with corresponding secondary antibodies at room temperature for 1 h. Specific protein bands were visualized with an ECL advanced Western blot analysis detection kit (Merck, Germany). Band intensity was measured by ImageJ software (NIH, Bethesda, MD).

### Statistical analysis

Results were expressed as means ± standard deviation (SEM) of at least three independent experiments. Statistical analyses were performed with GraphPad Prism 7 (GraphPad Software, San Diego, CA) and ImageJ 1.47v (NIH, Bethesda, MD). Results were considered significant if *p* < .05.

## Results

### The surface morphological structures of RSG/PHBV membranes

**Figure 2.**
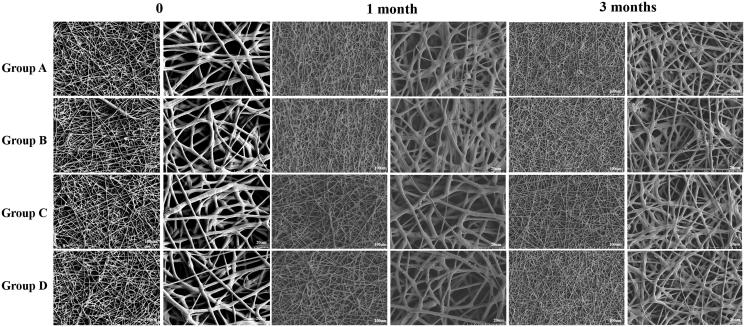
Morphological structures of RSG/PHBV membranes after 0 or 1 or 3 months. Magnification: left pictures of 0 or 1 or 3 months, 500×; right pictures of 0 or 1 or 3 months, 2000×. Scale bar: left pictures of 0 or 1 or 3 months, 100 μm; right pictures of 0 or1 or 3 months, 20 μm.

**Figure 3.**
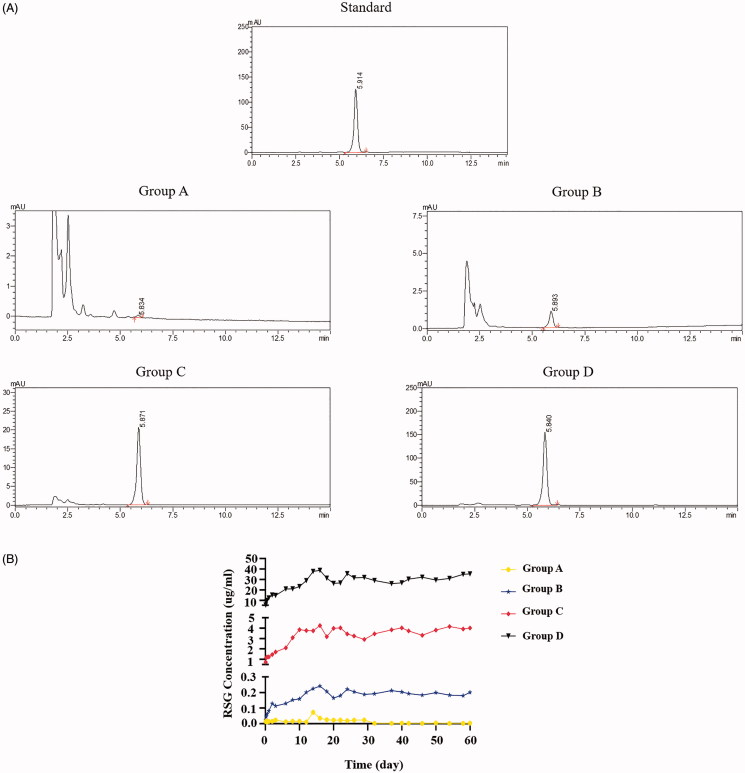
**(A)** A typical chromatogram of rosiglitazone (standard sample: 5.914 min), group A (5.834 min), group B (5.883), group C (5.871), and group D (5.840). **(B)** The RSG release curve of four groups.

SEM micrograph of RSG/PHBV membranes is shown in Figure 2. There are no significant differences among four groups in the morphology. The characteristic diameter of fibers was range from 5 to 50 μm and surface of the fibers was smooth. It demonstrated that the concentration of rosiglitazone would not affect the morphology of RSG/PHBV membranes. Next, we put the membranes into 6 cm dishes which contained 5 mL of physiological saline and then place them into the 37 °C incubator for 1 or 3 months to detect the behavior of degradation by SEM. Figure 3 shows the morphology of RSG/PHBV membranes after 1 month or 3 months. The surface of fibers became rough, the diameter of majority fibers became smaller and fibers became softer contrast to Figure 2. However, there are no differences of morphology change among four groups. Therefore, the degradation speed has no difference in four groups.

### The release behavior of RSG/PHBV membranes

The chromatograms of standard rosiglitazone, group A, group B, group C and group D are shown in Figure 3(A). The retention time of standard sample (rosiglitazone) was 5.914 min. The rosiglitazone release of group A, B, C, and D would detected around the retention time of standard. The RSG release curve was shown in Figure 3(B). The released concentration of RSG was associated with RSG final concentration which was contained in the RSG/PHBV film. The release peaks of RSG in group B, C, and D were all on the 16th day. RSG/PHBV membranes can persistently release RSG with 3 months at least.

### Bleb characteristics and survival curve

**Figure 4.**
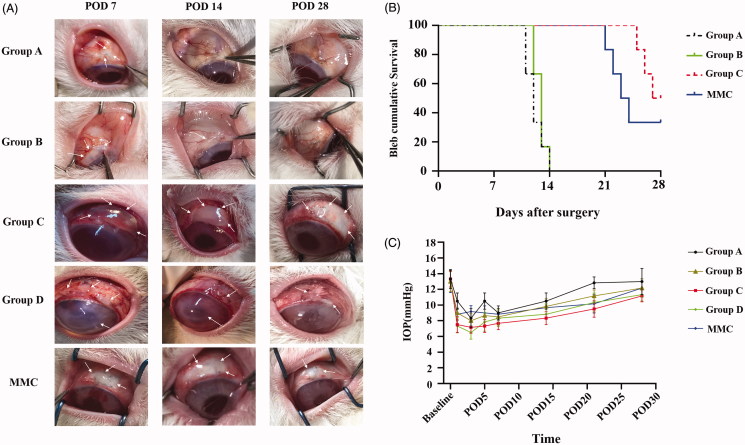
(A) Representative images show the appearance of filtration blebs in group A, group B, group C, group D, and MMC at POD 7, 14, and 28 after GFS. The white arrows in the pictures of group A, B, C, and MMC groups show the filtration blebs. The white arrows in the pictures of group D show the severe complications of conjunctiva, sclera and cornea. (B) Kaplan-Meier survival curve for group A, B, C, and MMC. (C) Graph shows the IOP elevation following GFS in group A, B, C, and MMC group.

We evaluated the effect of RSG/PHBV membranes treatment on the survival of filtering blebs following GFS. Figure 4 shows the appearance of filtration blebs in five groups on POD7, POD14 and POD28. Evaluation of the morphology of filtering blebs by IBAGS was summarized in Table 1. As shown in Figure 4(B), the bleb survival among the groups showed a significant difference for Kaplan–Meier survival curve (log rank; ****p < .0001). Moreover, the bleb survival times in the group C and MMC were significantly longer than that in the group A (blank control) (log-rank test; both, ***p = .0005). However, there was no significant difference in bleb survival time between the group C and MMC groups (*p* = .25). The Median survival time of group A, B, C, and MMC was 12, 13, 27.5, and 23.5 days, respectively. Group D (50 mg/mL RSG/PHBV membrane) caused tissue necrosis in both conjunctiva and sclera, severe corneal toxicity, and endophthalmitis. The exactly complications in five groups after GFS were shown in Table 2. So, we exclude this group in the following experiments. Taken together, these data suggest that RSG/PHBV membranes contained 5 mg/mL rosiglitazone treatment (group C) benefits the survival of filtering blebs following trabeculectomy and the beneficial effects appear to be greater than that of MMC.

**Table 1.** Indiana Bleb Appearance Grading Scale in these groups.

	POD 7	POD 14	POD 28
Group A	H1, E1, V2, S0	H0, E0, V1, S0	H0, E0, V1, S0
Group B	H1, E2, V3, S0	H0, E0, V1, S0	H0, E0, V1, S0
Group C	H3, E3, V4, S0	H3, E3, V3, S0	H1, E3, V2, S0
Group D	Not applicable		
MMC	H2, E2, V3, S0	H2, E3, V3, S0	H1, E1, V1, S0

H: Height, H0 (flat) to H4 (high); E: Extent, E0 (less than 1 clock hour) to E3 (more than 4 clock hours); V: Vascularity, V0 (avascular) to V4 (extensive vascularity); S: Siedel, S0 (no leak) to S2 (streaming).

**Table 2.** The number of different complications in five groups after GFS.

	Group A	Group B	Group C	Group D	MMC
Hyperemia	3/6	3/6	6/6	6/6	6/6
Chemosis of the conjunctiva	1/6	2/6	3/6	6/6	4/6
Corneal toxicity	1/6	0/6	2/6	6/6	3/6
Bleb leakage	0/6	0/6	0/6	2/6	1/6
Blebtitis	0/6	0/6	0/6	3/6	1/6
Endophthalmitis	0/6	0/6	0/6	1/6	0

# of animals with a complication/total # of animals.

### Intraocular pressure

IOP was measured before (baseline) and after surgery (day 1, 3, 5, 7, 14, 21, 28) at 10 AM. Figure 4(C) illustrated the IOP change after filtration surgery among the five groups. There was no significant difference in the baseline IOP before the surgery. In each group, IOP was reduced in the early stage after GFS and slowly increased with time. There are no significant differences between all the groups over the entire study period (*p* > .05) (Table 3).

**Table 3.** Summary of the mean IOPs (mmHg ± SD) in each group after GFS.

POD	Group A	Group B	Group C	Group D	MMC
0	13.33 ± 1.21	13.00 ± 1.27	13.33 ± 1.03	13.33 ± 1.21	13.00 ± 1.41
1	10.50 ± 1.05	9.00 ± 0.63	7.50 ± 1.05	7.33 ± 0.82	8.83 ± 0.75
3	8.33 ± 1.21	8.00 ± 0.89	7.17 ± 0.75	6.50 ± 0.84	9.17 ± 0.75
5	10.50 ± 1.05	8.67 ± 0.82	7.33 ± 0.82	7.83 ± 1.17	9.00 ± 0.89
7	9.00 ± 0.89	8.50 ± 1.05	7.67 ± 0.82	8.83 ± 1.03	8.83 ± 0.75
14	10.50 ± 1.05	9.83 ± 0.98	8.33 ± 0.82	8.83 ± 1.33	9.67 ± 0.82
21	12.83 ± 0.75	11.17 ± 0.75	9.50 ± 1.05	10.33 ± 1.21	10.17 ± 1.17
28	13.00 ± 1.67	12.17 ± 1.17	11.12 ± 0.75	11.33 ± 0.82	12.17 ± 1.17

### Histologic and Western blot examination on the fibrosis of bleb site

**Figure 5.**
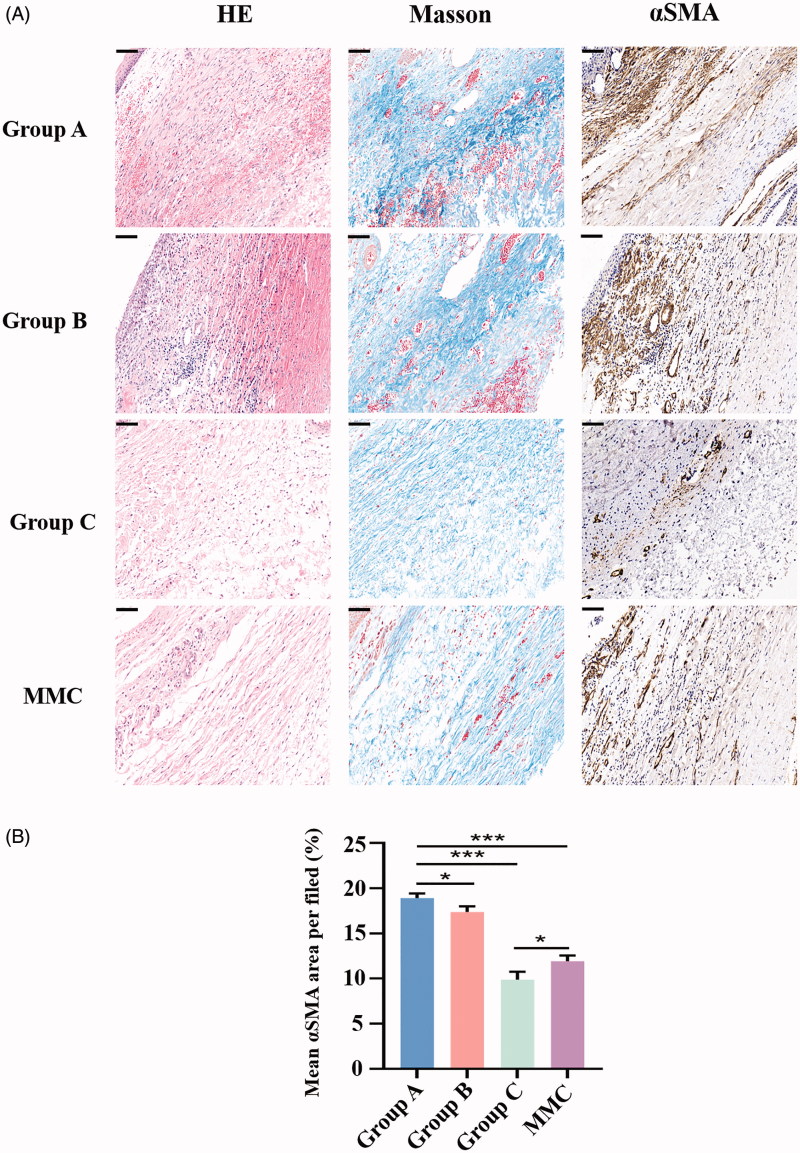
(A) Histologic features of each group 28 days after GFS. Sections were stained with HE, Masson’s trichrome and α-smooth muscle action immunohistochemistry. Magnification: ×300, Scale bar: 100um. (B) Graph shows the mean αSMA area per filed in group A, B, C and MMC. *p < .05, ***p < .001.

**Figure 6.**
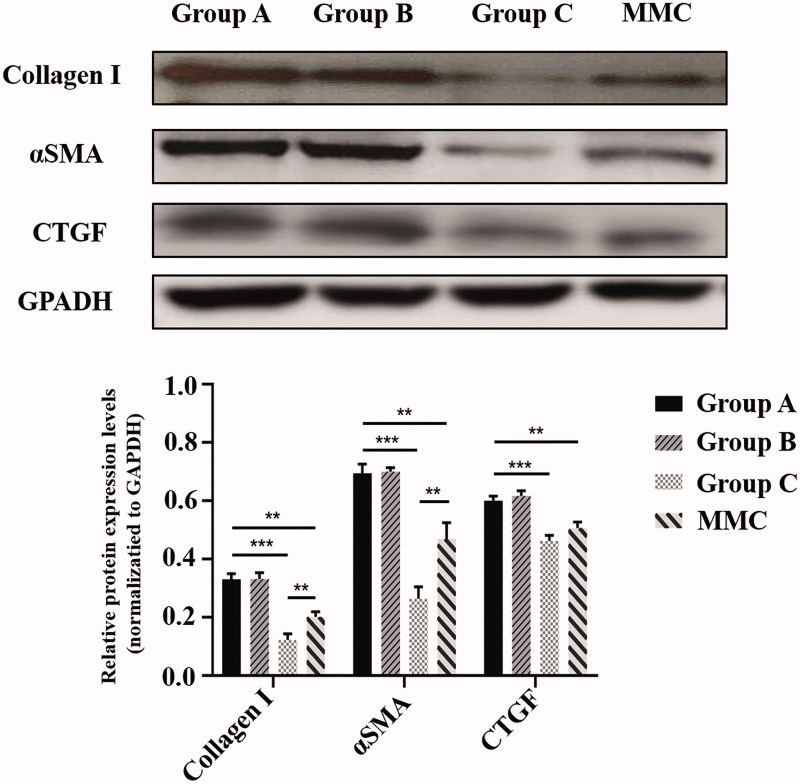
Rabbit bleb tissues in five groups were harvested at POD 28. Protein levels of Collagen 1, αSMA, and CTGF were determined by immunoblot. GAPDH was used as loading control. Immunoblots were scanned and the band intensity of each specific protein was normalized to that of corresponding GAPDH. Results are mean ± SEM (*n* = 3 per group). **p < .01 and ***p < .001.

To evaluate the subconjunctival fibrotic response to GFS-induced injury, HE staining, Masson’s trichrome staining and α smooth muscle actin (αSMA) (a marker of myofibroblasts) immunohistochemistry were conducted at POD 28 (Figure 5). Masson’s trichrome staining demonstrated reduced collagen deposition in the subconjunctival region in rabbits of group C and MMC, as compared to rabbits in group A. Moreover, the group C and MMC also showed the least proportion of fibrotic area compare with group A (both, ***p < .001). In addition, the mean fibrosis area per field of group C was significantly lower than that of MMC group (*p < .05). Next, we analyzed expression of Collagen I, αSMA, CTGF in bleb tissues isolated from rabbit eyes (Figure 6). Immunoblot analysis showed that protein levels of Collagen I, αSMA, and CTGF in group C were significantly attenuated contrast to group A (all, ***p < .001). Similarly, MMC treatment significantly attenuated GFS-induced expression of Collagen I, αSMA, and CTGF (all, **p <.01). In particular, group C has lower expression of Collagen I and αSMA compare to group MMC (both, **p < .01).

## Discussion

Since antimetabolite such as MMC and 5-FU have been increasingly used as an adjunctive to trabeculectomy and have become the first use to reduce scar formation in clinic. However, MMC was halted production in China since 2018. Majority patients i were accepted the use of 5-FU during the surgery. Unfortunately, most of them have to repeatedly injected 5-FU subconjunctivally after surgery because of the weak anti-scarring effect of 5-FU. That treatments enhanced the pains of patients. Therefore, there is a need for alternative strategies to prevent filtration failure for patients.

Sustained DDS is a usefully way to get stable, effective, long-term and safe drug treatment effects and increase patient compliance. Currently, DDS used in eye including eye implants (Xi et al., 2014), Nano drug release (Vicario-de-la-Torre et al., 2018), biodegradable polymers (Chan et al., 2019), electric pulse directed DDSs (Oshima et al., 1999), liposomes (Gai et al., 2018), gel (Gandhi et al., 2017), and so on. At the same time, DDS get more and more attention by many ophthalmologists. The study of Ozdemir et al. (2017), is to estimate uptake for a new technology that delivers sustained-release glaucoma medication and to investigate how uptake varies by product attributes, physician recommendations, peer adoption, and patient characteristics. The results of this study showed that the longer the interval between administrations the more likely it is that physicians would recommend it. Also, physicians would be likely to recommend it to patients with high income and to those with adherence problems. Therefore, the next study of sustained drug release should concentrate on the long-term effectivity and lower price.

PHBV has been a significant progress in the field of medical study. This polymer material is easily to get and harmless to environment in the produce process. Moreover, it is rather cheaper than other technologies. We combine rosiglitazone and PHBV by electrospinning. Similarly, rosiglitazone is a cheap medicine and easily acquired. Our study showed the application prospect of RSG/PHBV membrane and the use of electrospinning in medicine. Whereas, the long-term effectivity and safety of RSG/PHBV membrane need to observe in the next experiments.

## Conclusion

We conclude that RSG/PHBV membrane treatment prevents scar formation in a rabbit model of GFS by inhibition of profibrotic gene expression. Rosiglitazone release system represents a novel anti-scarring drug candidate to improve the success rate of GFS.
